# “Do Octopuses Have a Brain?” Knowledge, Perceptions and Attitudes towards Neuroscience at School

**DOI:** 10.1371/journal.pone.0047943

**Published:** 2012-10-17

**Authors:** Alessandra Sperduti, Federica Crivellaro, Paola Francesca Rossi, Luca Bondioli

**Affiliations:** Section of Anthropology, National Museum of Prehistory and Ethnography “L. Pigorini”, Rome, Italy; George Mason University/Krasnow Institute for Advanced Study, United States of America

## Abstract

The present study contributes to the question of school literacy about the brain, with an original survey conducted on Italian students from the 3^rd^ to 10^th^ grades (n = 508). The main goal was to test student's knowledge, attitudes, and interests about neuroscience, to assess needs, prospects, and difficulties in teaching about the brain from elementary to high school. A written questionnaire, maintaining anonymity, asked 12 close-ended multiple choice questions on topics related to human and animal brains, plus one facultative open-ended question about interests and curiosities on brain topics. The results show that respondents have a fragmentary level of basic knowledge about the brain, with aspects related to brain functions and consciousness the most challenging. As expected, degrees of performance improve with school level; elementary school students answered correctly an average number of 5.3 questions, middle school 6.5, and high school 7.4. Overall, students show great interest in the brain, as shown by the large number of questions gathered through the open-ended question (n = 384). Other topics are addressed, mostly related to brain structure/functions and the role of the brain in the everyday life. The survey indicates the need of more thorough school programs on this subject, reinforced by interdisciplinary teaching where comparative anatomy and evolutionary aspects of brain development are covered.

## Introduction

Since the “Decade of Brain” (1990-1999) brain literacy has become one of the primary goals for several neuroscience organizations, such as the Society for Neuroscience, the DANA Foundation, and the Italian Society of Neuroscience (SINS). New strategies for teaching and dissemination of neuroscience information are being proposed within specific formal and informal learning projects to fill the gap between science and society [1]–[5]. Indeed, in the last decades there has been a rapid development of brain research in its multiple themes and applications. Neuroscience has progressively gained a central role within the network of scientific studies and now represents a rather large hub, strongly interconnected with many other disciplines [6]. Furthermore, the newest lines of research and the use of more sophisticated techniques of analyses, such as neuroimaging, are adding data and insights that promote what some scholars name “neuroculture” [7], a term that underlines how pervasive neurosciences are becoming in the everyday life contexts (e.g. educational, medical, commercial, artistic, legal). The gap between the scientific development and the public understanding, though, is not easily bridged. Several authors have called for concrete actions to share the scientific achievements with a broader public [8], [9], and for the scientists to participate more actively in the current interdisciplinary debates about possible applications of the neurotechniques and their ethical implications [10]–[13]. However, the social sharing of knowledge about the brain is recognised as a complex task, further challenged by the risk of misinformation, inaccuracy or sensationalism on the scientific discoveries [14], [15]. For this reason, many scholars strongly believe that education about the brain should start at school in primary grades [5], [16]–[20]. It is argued this would create a greatly receptive environment to initiate new concepts and stimulating critical thinking [21]. To accomplish this, it is important to determine students' attitudes and knowledge for designing effective educational programs [22]–[25]. A few surveys aimed at measuring knowledge about the brain have been published thus far [1], [26]–[31]. They differ by goals, methods of inquiry, themes investigated, and the type of public addressed, but only a few of them deal specifically with pre-college students [22], [23], [25].

The present study contributes to the issue of knowledge about the brain in an original survey carried out with 508 Italian primary – high school students comprised of grades 3 to 10 (age 7 to 16).

## Methods

### Ethics statement

This study uses data that were collected anonymously and therefore any related information cannot be used individually. Moreover, during the survey no sensitive personal information was collected. The research project, its aims and modes of implementation were presented and discussed in detail with the headmasters and the teachers of the institutions that volunteered to participate. A verbal informed consent was obtained from the teachers on behalf of the students involved in this study. The students' parents were informed and gave their verbal consents for their children to participate to the survey. Verbal consents were not recorded, because the research involved normal educational practices. Moreover, such a verbal unrecorded consent was considered adequate because of the anonymous nature of the questionnaire, following Italian law 675/1996 and the subsequent Legislative Decree 196/2003. According to this same law (article 12, section 1, point d), all the information collected has been used solely for purpose of scientific research. The data collected in this research are also protected by statistical confidentiality and therefore can not be disseminated individually, but in summary form only (article 9 of Legislative Decree 6 September 1989, n. 322).

Our research project did not need approval from Italian ethics committee because there were neither clinical implications nor experiments with human subjects. The Italian legislation on the ethics committees (Ministerial Decree of 18 March 1998 concerning the guidelines of reference for the establishment and operation of ethics committees, published in OJ 122 of 28 May 1998) grounds on the World Medical Association Declaration of Helsinki – Ethical Principles for Medical Research Involving Human Subjects (JAMA, March 19, 1997-Vol.277, N°11, pg.925-926; http://www.wma.net/en/30publications/10policies/b3/index.html).

### Survey design

We designed a single written questionnaire for a survey across different grades of the Italian pre-college school, from elementary to high school (see Table S1). A total of 508 Roman students participated to the survey, of which 217 came from elementary school (from grade 3^rd^ to 5^th^), 139 from middle (from grade 6^th^ to 8^th^), and 152 from high school (from grade 9^th^ to 10^th^). The overall sample totals 243 females, 251 males and 14 individuals of unknown gender (missing information), evenly represented across all the school grades. The questionnaire (Text S1) was organized in four sections. The first section collected individual background information, i.e. gender, age, school grade, whether the student recently attended any class covering topics about the brain, and if he/she owned a dog or cat. The second section contained 12 close-ended multiple choice questions on brain functions and cognitive abilities of humans and other animals. For each question it was specified whether to select a single answer or more than one. In the third section, the students were asked to assess the level of difficulty of the test. The last section asked the students to ask their own questions and list their curiosities about the brain, on a space limited to half page. The time limit to fill in the questionnaire was 45 minutes.

### Statistical analysis

All data were analysed by contingency tables for discrete traits and descriptive statistics for continuous ones. The interaction between factors in the contingency tables was estimated by the chi-squared test. Probability values were scored for three levels of significance, p <0.001 very highly significant, p<0.01 highly significant, and p <0.05 significant. If at least one contingency table cell showed an observed count of less than 5, the p-value was calculated by Monte Carlo simulation based on 10,000 replicates. Analysis of variance was used to test the difference among the means of the quantitative variables. The significance of the interaction between levels was tested by the Tukey's Honest Significant Difference test [32]. All the analyses were performed with the R package (version 2.15.0) [33].

## Results

**Figure 1:**
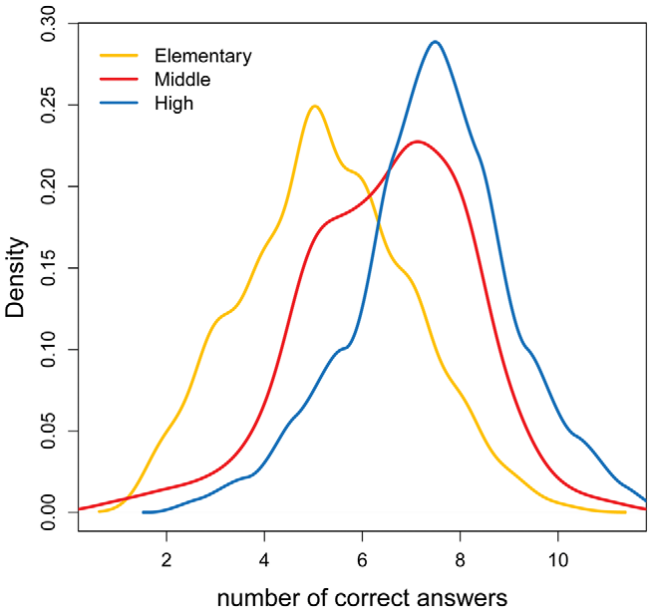
Density plot of the distribution of the number of correct answers by school level.

All students (n = 508) completed the questionnaire within the time limit. Their level of performance was calculated for the number of correct answers on the 12 questions of section 2 of the questionnaire. Overall, the average number of correct answers was 6.3 (sd = 1.9), ranging from 1 to 11. The highest score (11/12) was reached only by five respondents, none from the elementary grades. We tested whether the level of performance depended on: school level, sex, having recently studied the brain, and the students' rating of the overall level of test difficulty. The only significant difference between means was found with respect to the three school levels (elementary 5.3, sd = 1.7; middle 6.5, sd = 1.7; high 7.4, sd = 1.6; one-way Anova F = 77.04, p<0.001). Tukey's test reveals that all means differ significantly from each other. The kernel density estimates of the sample distribution by number of correct answers are presented in Figure 1. Table 1 reports the results of the performance per school level.

**Table 1:** Table 1. List and abbreviations of the close-ended questions.

		Total	Elem.	Mid.	High	
	Number of students	508	217	139	152	

	List of questions provided in the questionnaire	*% of correct answers*				
Q1	*What is the brain for?*	16.7	8.8	16.5	28.3	p<0.001
Q2	*What is your brain doing now?*	5.5	6.5	5.0	4.6	n.s.
Q3	*Do the different parts of the brain perform different functions?*	89.8	79.7	95.0	99.3	p<0.001
Q4	*The brain is mostly made of…*	84.1	78.8	90.6	85.5	p<0.01
Q5	*What makes you feel fear?*	70.7	53.0	79.1	88.2	p<0.001
Q6	*What makes you act courageously?*	76.4	66.8	81.3	85.5	p<0.001
Q7	*How have you learned what you know and can do today?*	18.7	8.3	17.3	34.9	p<0.001
Q8	*Only humans have a brain?*	97.8	98.2	95.0	100	p<0.05
Q9	*What other living species do have a brain?*	63.8	56.2	63.3	75.0	p<0.01
Q10	*A cat or a dog is able to…*	25.6	16.6	30.9	33.6	p<0.001
Q11	*What does it mean to have consciousness*	49.6	30.9	48.2	77.6	p<0.001
Q12	*Which of the following has consciousness*	26.6	24.0	28.8	28.3	n.s.

Percentages of correct answers and level of significance are shown by school level (n.s.  =  not significant).

### Human brain functions

**Figure 2:**
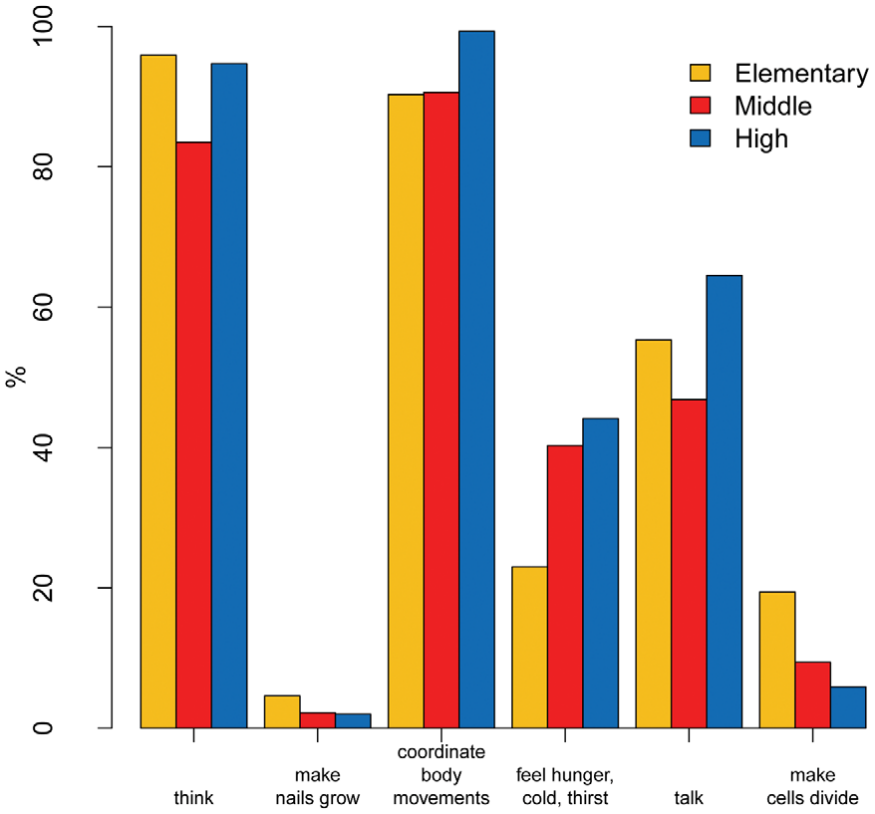
Bar plot of the answers to Q1 “*What is our brain for?*” by school level.

Student knowledge about general human brain functions was tested by three questions (Q1, Q2, Q3; Table 1). The first of these “*What is the brain for?*” offered the following possible answers, namely: 1. *thinking*; 2. *making nails grow;* 3. *coordinating body movements;* 4. *feeling hunger, thirst, cold;* 5. *talking;* 6 *dividing cells.* The students were asked to choose more than one answer. The correct answering pattern was considered 1, 3, 4, and 5. Only a low percentage (16.7%; N =  508) selected the answers accordingly, which correlates significantly with school level (elementary school 8.8%; middle school 16.5%; high school 28.3%; p<0.001). In fact, while most of the students recognised that the brain has the function of *thinking* (90.2%) and *coordinating body movements* (93.1%), many did not include other functions such as *talking* (only 55.7%) and *sensing hunger, thirst, cold* (34.1%) (Figure 2).

The second question “*What is your brain doing now?*” was intended to test student knowledge that their brains can perform multi-tasking. The choices provided were: 1. *reading and understanding the questions*; 2. *dreaming;* 3. *thinking how to answer;* 4. *making your heart beat;* 5. *making your hand move;* 6. *regulating your body temperature.* We considered the correct answering pattern as 1, 3, 4, 5, and 6, which was given by only the 5.5% of the whole sample. Regardless of school grade, most students seemed to be unaware that the brain is in fact responsible for body functions involving heart functioning and thermoregulation, with only 21.7% and 12.0% ticking these options, respectively.

The third question asked if different parts of the brain perform different functions, or whether they are all the same. Most of the students answered correctly (89.8%) with a significant increase in performance associated to age (elementary 79.7%; middle 95.0%; high 99.3%; p<0.001).

### Brain composition

The question “*The brain is mostly made of…*” (Q4; Table 1) was to be completed with only one of the following options: 1. *skin*; 2. *neurons*; 3. *muscles*. The level of performance was generally high across all school levels, with the 84.1% of the respondents choosing the right answer “*neurons*”. The performance varies significantly across the three levels (p <0.01), with students from the elementary school being the weakest (78.8%), middle school ones showing the best scores (90.6%), and high school students being intermediary (85.5%). The pairwise comparison between high and middle levels is not statistically significant. This datum is not positively correlated with whether or not students have recently attended classes on brain morphology and functions.

### Emotions and decision making

**Figure 3:**
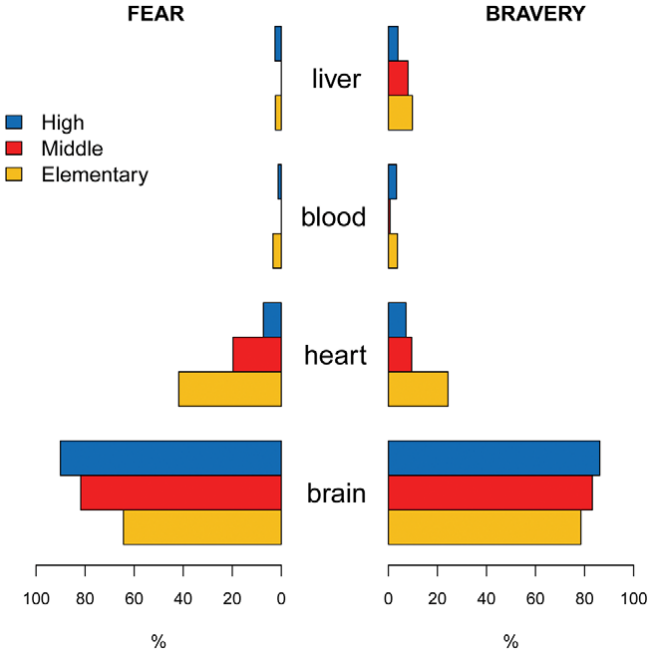
Double bar graph contrasting the students' answers to Q5 “*What make you feel fear*” and Q6 “*What make you act courageously*”, illustrated by school level.

We also investigated whether young people associate emotions and decision making processes with cerebral activities. We asked two interrelated questions: “*What makes you feel fear?*” and “*What makes you act courageously?*” (Q5 and Q6; Table 1). In both cases, the students were asked to choose only one answer among: 1. *the blood;* 2. *the heart;* 3. the *liver;* and 4. *the brain.* Respectively, 70.7% and 76.4% of the total sample answered correctly, with older students performing significantly better than younger ones (p<0.001). In the particular case of fear, elementary students failed almost four times more than high school ones (47.0% vs. 11.8%). Errors were mainly associated to the choice of “*the heart*” in the first question (24.6%), and “*the heart*” or “*the liver*” in the second question (15.0% and 7.5% respectively) (Figure 3). A combined analysis of the two questions reveals a statistically significant association (p<0.001), with the majority of the respondents answering correctly to both (63.5%).

### Learning

The questionnaire tested the capability of the students to recognize different ways of learning (Q7; Table 1). The question asked “*How have you learned what you know and do today?*”. Four possible choices were provided: 1. *I have watched others;* 2. *Everything was already inside in my brain;* 3. *I was taught;* 4. *I learned on my own.* The correct answering pattern included choices 1, 3, and 4, which was achieved by only 18.7% of the students. This result is positively correlated with the students' age (p<0.001), with only 8.3% of the elementary, 17.3% of the middle school, and 34.9% of the high school students answering correctly. The majority of the remaining answers were incomplete rather than incorrect. In fact, the analysis per single option reveals that while most respondents (91.7%) recognise the importance of being taught in the learning process. A smaller proportion are aware that they can learn by observing the others (62.2%) and by self-discovery (27.4%). The idea that we were born knowing everything already was accepted by only 3.4% of the respondents.

### Brain of other living species

**Figure 4:**
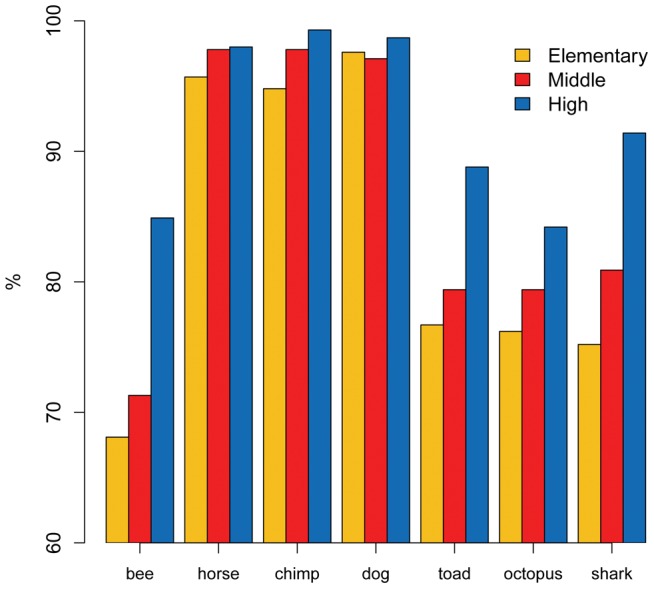
Bar plot of the students' answers to Q9 “*What living species do have a brain*”, illustrated by school level. The graph shows data related to animals only.

Do students know that other animals also have brains or that plants lack them? This was investigated through two different questions (Q8 and Q9; Table 1). The first asked whether humans are the only ones to have a brain, and rightly the majority of students disagreed (97.8%). The following question asked what other living species have a brain? Possible choices were: 1. *a bee*; 2. *an oak*; 3. *a horse*; 4. *a chimpanzee*; 5. *a dog*; 6. *a toad;* 7. *an octopus;* 8. a *shark;* 9. *a mushroom*. We scored only one combination of answers as the correct pattern, i.e. all but choice 2 and 9. As expected, the older the student, the higher the correctness of the responses (elementary  = 56.2%; middle  = 63.3%; high-school  = 75%; p<0.01). The students excluded almost unanimously that plants and mushrooms may have a brain, and the great majority agreed that mammals (dog, chimpanzee, horse) have one. More doubts arose about whether other vertebrates (toad, shark) as well as invertebrates (bee, octopus) may have brains (Figure 4).

### Capabilities of cats and dogs

To further inquire about the students' knowledge and perception of the brain capacities of the other animals, the tenth question asked to complete the following statement: “*In your opinion, a cat or a dog is able to…*” with more than one of the following options: 1) *learn;* 2) *speak;* 3) *remember;* 4) *think;* 5) *communicate;* 6) *feel pain;* 7) *dream*. The correct answering pattern included all but choice 2. We decided to include the choice “*dream*”, although we are aware that at present it is unknown if non-human animals actually dream as humans understand a dream. On a total of 508 answers, only 130 students chose the complete correct pattern (25.6%), with different other combinations given variably. Overall, we observed a general tendency to underestimate the capacities of these animals, with a large number of students excluding that they can “*think*” (47.0%) or “*dream*” (37.2%), but also “*communicate*” (21.5%) or “*remember*” (17.7%). Because of greater familiarity with their pet's habits, we expected those having a dog or cat at home would be more accurate in answering this question. Surprisingly, they did not do significantly better than those without (29.1% owners vs. 29.9% non owners). The correlation was run on a subsample of 372 students, being the number of those specifying whether they had a pet at home.

### Consciousness

**Figure 5:**
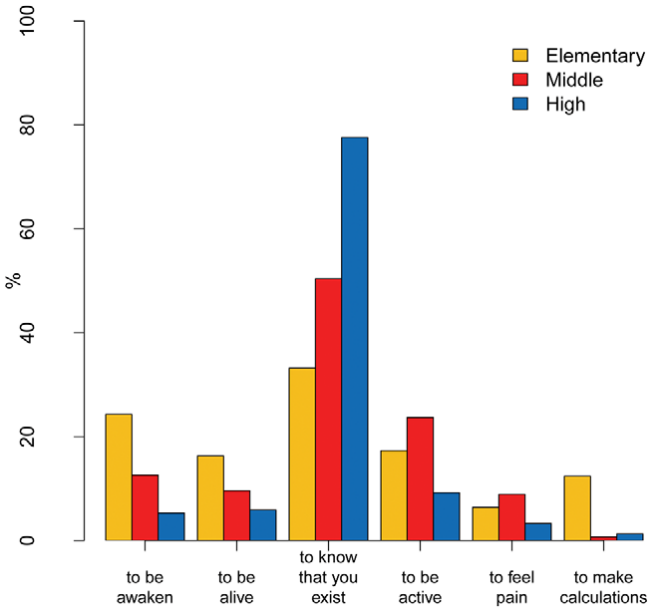
Bar chart illustrating by school level the answers to Q11 “*What does it mean to have consciousness?*”.

The last two questions dealt with consciousness (Q11 and Q12; Table 1). The first, “*What does it mean to have consciousness?*”, with six choices: 1. *to be awake*; 2. *to be alive*; 3. *to know you exist*; 4. *to be active*; 5. *to feel pain*; 6. *to know how to do math*. The only correct answer was 3, which was given by the 49.6% of the respondents. The level of performance positively correlates with school levels (p<0.001), with a rather low percentage for the elementary school students (30.9%) and a much higher one for the middle school (48.2%) and for the high school ones (77.6%). The students' choices are shown in the bar graph in Figure 5.

The twelfth question queried who/what has a consciousness: 1. *you*; 2. a *computer*; 3. a *mushroom;* 4. a *Neanderthal*; 5. a *snail*. About one quarter (26.6%) of the students chose the right combination of answers (1 and 4), with no significant difference among the three levels. However, the analysis by single choices shows that almost the totality of the respondents assess that they have consciousness (97.4%), whereas only about half of the students recognise that Neanderthals did (55.9%). Surprisingly, quite a high proportion attributes consciousness to snails (34.8%). Also, 19.4% of primary students and 6.6% of middle school ones attribute consciousness to computers. The combined analysis of Q11 and Q12 reveals that knowing what consciousness is does not predict knowing who has it. Only one third (31.3%) of those answering correctly to Q11 attributes consciousness to both us and the Neanderthals.

### What students want to know about the brain

The last section of the questionnaire presented an open question aimed to collect the students' interests, curiosities, and doubts about the brain and related topics. 261 students provided a total of 384 responses. As shown in Table 2, a variety of topics are explored, the majority further inquiring on the brain structure and its functions. Some of the questions have been stimulated by the questionnaire. Others are some original inquiries mostly related to the subject of thinking and intelligence, memory, sleep and dream, individual and male/female brain differences. Health and methodological issues are of less interest and are mainly asked by high school students. Only a small proportion of the students (mainly from primary school) queried about the evolution of the human brain and its development.

**Table 2:** Summary table of the students' curiosities collected through the open question “*What else would you like to know of the brain?*”, grouped by topics.

	Total	Elem.	Mid.	High
*N of students*	508	217	139	152
*N of students asking at least 1 question*	261	119	78	64
*N of total questions*	384	173	120	91

Topics of interest	*% on the total questions*			
general brain functions	15,5	14,5	15,3	17,6
general brain anatomy	15,2	21,4	14,4	4,4
animal brain	9,9	9,2	4,5	17,6
Intelligence	7,5	10,4	3,6	6,6
consciousness	6,7	5,2	9,9	5,5
Memory	4,5	3,5	7,2	3,3
sleep/dream	4,5	2,3	5,4	7,7
the brain and body movements	4,5	6,9	3,6	1,1
Neurons	4,0	4,6	2,7	4,4
individual differences	3,7	2,3	3,6	6,6
use of only 10% of the brain	3,7	0,0	9,0	4,4
brain in the prehistory	3,5	5,2	1,8	2,2
brain growth and development	3,2	5,8	0,9	1,1
methods and techniques of brain studies	2,9	1,7	2,7	5,5
health and disease	2,7	1,2	1,8	6,6
Others	8,0	5,8	13,5	5,5

## Discussion

The results show that basic brain knowledge across our sample is fragmentary. Indeed, most of the answering patterns were more often incomplete than totally wrong, in particular for Q1, Q2, Q7, Q9, and Q10 (see Table 1). The results show that performance increases with school level. As expected, some degrees of overlap in the distribution of correct answers is observed across the sub-samples (Figure 1). Higher grade students generally show better critical assessment on more complex subjects such as what consciousness is (Q11), or in recognising different ways of learning (Q7). A few questions were more challenging overall, scoring less than 20% of correctness, such as Q1, Q2, and Q7, which were incomplete rather than mistaken. Only three questions scored more than 80% of correctness, namely Q3, Q4, and Q8, the least demanding also because of the reduced number of choices provided. Nevertheless, a comparison with the data reported in Herculano-Houzel [27] shows that the present Italian sample scores similarly to the Brazilian high school students on questions related to the distinctive functions of different brain parts (Q3) and to the relationship between brain and emotions (Q5). Still, elementary school students from our sample show more difficulties on this latter subject, as shown also by the scoring of Q6 (“*What makes you act courageously?”*). Such errors can be interpreted as misconceptions derived by typical Italian idioms such as “*having liver*”, “*having heart*”, “*to maintain a cold blood*” for having courage; or “*having the heart beating*”, “*to feel the blood freezing in the veins*” for feeling fear.

The students show an average level of knowledge for the presence of a brain in other animals (Q9) and about animal cognitive capacities (Q10). Whether they easily assess that species phylogenetically closer to *Homo sapiens* have a brain, they fail to acknowledge the presence of this organ in the more distantly related taxa, such as octopus, toad, bee, and shark (see Figure 4). The students tend to underestimate cognitive capacities of dogs and cats, often excluding faculties such as dreaming, reasoning, communicating, and remembering. Furthermore, as shown above, having a dog or cat does not increase the frequency of correct answers. These results have no direct comparison in the literature. In fact, Herculano-Houzel [27] tested different aspects of animal brain knowledge, focusing on the relationship between body size, brain size and intelligence, and showed that both neuroscientists and the general public have no clear understanding about the relation between body size and brain size across the different animal species.

The results from our school sample, together with the Brazilian one, show that teaching neuroscience is confined to human anatomy, lacking of any basic comparative approach with the nervous systems of other animals. Conversely, scientists today consider this of fundamental importance to better understand the adaptive and evolutionary processes underlying brain differentiations, as well as the evolution of higher properties unique to our species [34], [35], [36]. In this respect, the deeply debated topic of consciousness was here explored through two related questions (Q11 and Q12), the latter inquiring who else has self-awareness. Strikingly, most of the students erroneously excluded Neanderthal, but included the snail in their answers!

In order to better design neuroscience educational programs, besides assessing the students' literacy level, it is also fundamental to define their interests and motivations, as well as to know what topics they are particularly curious about. Hence, the information collected through the final open-ended question “*What else would you like to know of the brain?*” is of key interest. It may well help promoting a “pull approach” in education (i.e. what the public is interested in) instead of the “push” approach based on what we suppose the public should know. This kind of survey is being applied more and more by science educators with important outcomes that should not be neglected [28], [37]–[40]. Instead, they should be exploited to open dialogues in which the public's curiosities are used as portals to introduce themes that scientists and educators deem important. The large number of questions gathered shows that the students of our sample are greatly interested in the brain subject. They are mostly curious of topics related to the brain structure and functions, and the everyday life (such as intelligence, memory, individual differences). Questions like “*What else does the brain do?*” and “*How does the brain work?*” were frequently asked by the respondents. Little interest was shown towards health and medical aspects of the brain, similar to results observed in other school and public surveys [28], [37]. Some of the students' questions reveal misconceptions, such as the possibility that the brain may have superpowers and the conviction that we use only the 10% of our brain (stated out in 14 free questions). Herculano-Houzel [27] recorded similar evidence on a sample of high school, college and post-graduate students. Such a misconception seems to be a deep rooted ‘neuromyth’ in our society [41].

### Conclusions

This study provides new data about the level of ‘brain literacy’ on a large sample of Italian pre-college students. Besides testing their knowledge on basic brain facts, it also investigated their capacities to observe reality, to deal with complex thinking, and to self-interrogate about brain related topics. In Italy, teaching programs foresee the study of the human brain as part of a broader knowledge of human anatomy, which is taught at different levels across all school years. Nevertheless, high school students declare to have little knowledge about it despite the interest shown towards the discipline [42]. Indeed, our study confirms this fact and shows that pre-college students have a fragmentary knowledge on these subjects, notwithstanding their interest and curiosity. Our results show that neuroscience is still taught by focusing merely on anatomical and basic functional aspects of the human brain, lacking of any comparative and interdisciplinary perspectives that would enable a more comprehensive understanding of the evolution of the brain across time, and the definition of what we share with other animals versus what makes us unique as humans. Aspects derived from comparative neuroscience and palaeoneurology [43], [44], two lively branches of neurobiology and human evolutionary studies, should be better covered in school.

## Supporting Information

Table S1 **Summary of the sample composition per school grade.** This study uses US school grade standards (first column) which correspond to the Italian grades illustrated in the second column.
(PDF)

Text S1 **The questionnaire “Open Your Mind!”.**
(PDF)
