# Supplementary material for: “Do Octopuses Have a Brain?” Knowledge, Perceptions and Attitudes towards Neuroscience at School
Source: PLoS One. 2012 Oct 17;7(10):e47943. doi: 10.1371/journal.pone.0047943 (PMC3474737; doi:10.1371/journal.pone.0047943)
Supplement: Table S1 — Summary of the sample composition per school grade. This study uses US school grade standards (first column) which correspond to the Italian grades illustrated in the second column. (PDF) [file pone.0047943.s001.pdf]

“DO OCTOPUSES HAVE A BRAIN?” KNOWLEDGE, PERCEPTIONS AND ATTITUDES TOWARDS NEUROSCIENCE AT SCHOOL

Alessandra Sperduti, Federica Crivellaro, Paola Francesca Rossi, and Luca Bondioli

Supplementary Information Table 1

Summary of the sample composition per school grade. This study uses US school grade standards (first column) which correspond to the Italian grades illustrated in the second column.

Grade	Italian Grade	Age Range	Males	Females	Unrecorded	Total
3rd (elementary)	III elementare	7-9 y	16	10	9	35
4th (elementary)	IV elementare	8-10 y	62	65	1	128
5th (elementary)	V elementare	9-11 y	31	20	3	54
6th (middle sch.)	I media	10-12 y	6	12	-	18
7th (middle sch.)	II media	11-13 y	27	30	-	57
8th (middle sch.)	III media	12-14 y	30	32	2	64
9th (high sch.)	I superiore	13-15 y	25	39	2	66
10th (high sch.)	II superiore	14-16 y	51	35	-	86
					**TOTAL**	**508**
