# Supplementary material for: “Do Octopuses Have a Brain?” Knowledge, Perceptions and Attitudes towards Neuroscience at School
Source: PLoS One. 2012 Oct 17;7(10):e47943. doi: 10.1371/journal.pone.0047943 (PMC3474737; doi:10.1371/journal.pone.0047943)
Supplement: Text S1 — The questionnaire “Open Your Mind!”. (PDF) [file pone.0047943.s002.pdf]

Ministero per i Beni e le Attività Culturali

SOPRINTENDENZA AL MUSEO NAZIONALE PREISTORICO ED ETNOGRAFICO "*L. PIGORINI*"
ROMA

Sezione di Antropologia

"Open your mind!"

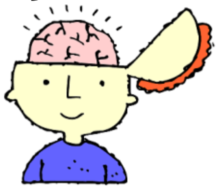

Date ______________

School name ____

Grade _____________________________ **Sex:** **Female** **Male**

Have you studied the brain recently? ☐ YES ☐ NO

Do you have a cat or a dog at home? ☐ YES ☐ NO

1. What is the brain for? *Choose **more than one** answer*

√ thinking
☐ making nails grow
√ coordinating body movements
√ feeling hunger, thirst, cold
√ talking
☐ dividing cells

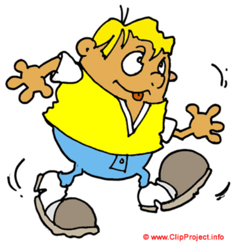

2. What is your brain doing now? *Choose **more than one** answer*

√ reading and understanding the questions
☐ dreaming
√ thinking how to answer
√ making your heart beat
√ making your hand move
√ regulating your body temperature

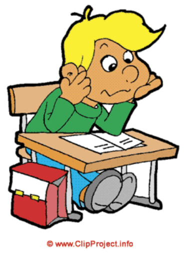

3. Choose the right assessment:

√ The brain is divided in several parts that perform different functions
☐ Each part of the brain is equal, all performing the same functions

4. The brain is mostly made of… *Choose **only one** answer*

☐ skin
√ neurons
☐ muscles

5. What makes you feel fear? *Choose **only one** answer*

☐ the heart
☐ the blood
☐ the liver
√ the brain

6. What makes you act courageously? *Choose **only one** answer*

☐ the heart
☐ the blood
☐ the liver
√ the brain

7. How have you learned what you know and do today? *Choose **more than one** answer*

√ I have watched others
☐ Everything was already inside my brain
√ I was taught
√ I have learned on my own

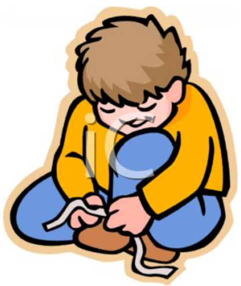

HUMANS AND OTHER ANIMALS

8. Only humans have a brain? ☐ YES √ NO

9. Among the following, who has a brain? *Choose **more than one** answer*

√ a bee ☐ an oak √ a horse √ a chimpanzee √ a dog
√ a toad √ an octopus √ a shark ☐ a mushroom

10. In your opinion, a cat or a dog is able to: *Choose **more than one** answer*

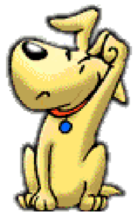

Learn	√ YES	☐ NO
Speak	☐ YES	√ NO
Remember	√ YES	☐ NO
Think	√ YES	☐ NO
Communicate	√ YES	☐ NO
Feel pain	√ YES	☐ NO
Dream	√ YES	☐ NO

11. What does it mean to have consciousness? *Choose **only one** answer*

☐ To be awake
☐ To be alive
√ To know you exist
☐ To be active
☐ To feel pain
☐ To know how to do math

12. Based on your previous answer, choose who/what among the following could have consciousness. *You may choose **more than one** answer*

√ you
☐ a computer
☐ a mushroom
√ a Neandertal
☐ a snail

Was the questionnaire difficult?

☐ Very difficult ☐ Difficult ☐ Easy ☐ Very easy

Write below all your curiosities and doubts about the brain. What else would you like to know of the brain? What is not clear to you?
